# Letters on Pulmonary Phthisis, Delivered in Jervis-Street Hospital; Comprehending the Pathology, Diagnosis, and Treatment of the Disease, with an Appendix

**Published:** 1846-07

**Authors:** 


### Art. VII.

*Lectures on Pulmonary Phthisis, delivered in Jervis-street Hospital; comprehending the Pathology, Diagnosis, and Treatment of the Disease, with an Appendix.* By J. T. Evans, m.d., &c.—*Dublin*, 1844. 8vo, pp. 196.

This is a curious book in more respects than one. It is written by a member of the profession who formerly "spent the greater part of his time for some years in assisting St. John Long in his daily frictions;" and who now proclaims that, "after twelve years of regular medical study, he does not think the time that he was thus so strongly engaged *by any means misspent*." (p. 131.) What this author may have gained either in an intellectual or moral point of view during the period he refers to, neither appears on the face of things, nor is stated in his work. On the other hand, he, in the page following that which contains the above declaration (p. 132), characterises his past conduct as "*a reckless trifling with human health and life;*" whence it should follow, but we believe it does not follow, that, in the estimation of Dr. J. T. Evans, time passed in a reckless trifling with human health and life is not by any means misspent. It is highly probable that St. John Long held the same creed, until the death he had been so active in dealing unto others paralysed his arm for ever.

The author is perfectly aware that multiplication of books is an evil; consequently, "if the present work did not contain some things which he believed to both novel and useful, it should never have been offered to the public." As the "best apology" for bringing it before the profession, the writer alludes in the preface to "*some* of the *most important and original*" views discussed in his pages. We proceed to attend him in his references to these views.

The reader is first informed that "the presence of tubercles appears, in the opinion of authors, to constitute the essence of the disease,"—a notion which Dr. Evans is earnest in proving to be hollow and erroneous. If Dr. Evans will produce evidence that one single author of repute teaches the doctrine he here ascribes to the class, we will acknowledge there is here something more than a rather worn-out artifice of gentlemen desirous of notoriety as reformers. Does Louis teach the monstrous absurdity which he is here stigmatized as promulgating? No; he simply tells us that tubercle constitutes the anatomical character of phthisis. Does Carswell's voice give justification to that error? Far from this; it announces the blood to be diseased from the first in phthisical subjects, and this before the development of local tuberculization. Does Clark, in his practical volume, give any colour of justice to the accusation of Dr. J. T. Evans? Less than all others even; for in the state which he styles "tuberculous cachexia," a state fully evolved before the generation of pulmonary tubercle, he recognizes the physical substratum of the disease. But if Dr. J. T. Evans has less of the character of an original teacher here than he would desire to persuade the pupils of the Jervis-street Hospital, we willingly concede him the merit of perfect unconditional originality in the announcement that "tubercles exert little or no influence on the progress of phthisis!" This is something new with a vengeance. What! tubercles which lead to inflammation of the pulmonary substance, pleuritis, perforation of the pleura, pneumo-thorax, pleuritis sometimes accompanied with agonizing pain, in itself capable of shortening existence,—tubercles which block up and render useless or actually destroy (it may be) three quarters of the entire pulmonary substance,—which cause a reduction *pro tanto* of the oxygenating surface for the vivification of the blood,—which are followed by the obliteration of much of the old, and the generation of a new, vascular system in the lung,—which lead to the development of similar tubercles in the meninges, a development that may cut off individuals in a few days,—which cause ulceration of the intestinal surfaces, ulceration that by its very cure sometimes destroys life, and by its propagation renders existence torture,—what! tubercles, which do all this, exert no influence on the progress of phthisis! Verily, there is novelty and genius here; and when Dr. Evans proves his thesis, none but persons, in whom jealousy knows no limit, will dream of contesting his right to an excessively capacious niche in the Temple of Fame.

The next discovery made by the author is that "morbid anatomy is not pathology,"—a novelty absolutely of the first water, and which is about of the same character as the following:—healthy anatomy is not physiology: whereof we make Dr. Evans free gift for his next course of lectures in Jervis-street.

Dr. Evans objects to the division of the disease into two stages, as totally insufficient; and has divided the disease into several stages, corresponding to what he believes to be the progress of the *lesions*,"—a word, by the way, not English in the Gallic sense in which it is here employed. The proceeding seems somewhat inconsistent for a person who regards the main of these *lesions*, namely, tuberculous matter, as a matter of no consequence. But *aliquando bonus Homerus*—and why should not Dr. J. T. Evans be entitled to his occasional snooze?

The next novelty is the reference of several of the ordinary symptoms of phthisis, "especially *those of impeded circulation*, to the condition of induration, the result, as I believe, of chronic pneumonia, which so frequently takes place in the neighbourhood of tubercular masses or of cavities." Heaven help our ignorance; we had fancied that, since Dr. Carswell demonstrated the connexion of the said impeded circulation and chronic pneumonia, the point was common property. But here Dr. Evans lays rude hands on the bantling, and affirms he is its father. We simply demur until he shall have proved his paternity.

Dr. Evans holds that, in M. Fournet's book, there are "many *old observations* put forward with an imposing air of novelty, and many *new assertions* destitute of foundation,"—an opinion we should not have thought it necessary to notice, except from its furnishing, we would humbly suggest, a singularly appropriate epigraph for Dr. Evans's own second edition. This stern pathologist and modest lecturer considers M. Louis' "etiology illogical, his diagnosis rather meagre, and his treatment inadequate;" but, probably fearful of blasting the French author's reputation utterly, Dr. Evans condescends to observe, "*I* cannot but thank M. Louis for the candour and truthfulness of his observations." (p. 192.) Happy the man who receives thanks from so eminent a quarter: it is truly comforting to know that, after one may have learned to think lightly of having enjoyed the highest European reputation for some dozen years, and felt that even that earthly good is tainted with evil, a vote of thanks may come from Dr. Evans to renovate the delightful feelings of early success.

Turn we next to the author's lectures on treatment,—a chapter which might be expected, in consideration of his early occupations, to be particularly instructive. These lectures open with various flourishes concerning "fond and devoted parents,"—rescuing victims from "the dark recesses of the lone and silent tomb," &c. &c., and next proceed to show that the profession has not advanced a step towards improved treatment of the disease since the days of Celsus. Anon the lecturer displays in learned phrase the folly of persons who treat symptoms of the same name always by the same remedies, and illustrates the said folly by sundry examples. Take one of these, and *ab uno disce*—"The *night-sweats* may depend on the rapid circulation of the fever of emaciation occurring in the relaxed condition of deficient growth; or they may be critical to the repeated forms of intercurrent pneumonias, tuberculization, and suppuration already referred to; or they may be vicarious to the natural discharges from other surfaces, as when they are accompanied by constipation." (p. 97.) Now, we very candidly confess our inability to comprehend what is meant by "the rapid circulation of the fever of emaciation occurring in the relaxed condition of deficient growth,"—so that not for worlds would we dispute the reality of the influence which Dr. Evans ascribes to the thing, whatever it is. Secondly, we deny that any human being has ever proved, or even in any remote degree made it probable as a matter of fact, that the night-sweats of phthisis are "critical" to intercurrent pneumonias or other of the states mentioned. Thirdly, it has clearly been shown by Louis,* that the notion of the vicariousness of sweats in the disease is a mere fallacy, the offspring of fancy and not of observation.

Dr. Evans proceeds to inquire, "what, in fact, are the lesions in which phthisis pulmonalis necessarily consists?" The response informs us that among these "lesions" rank, for example, "diminished force of growth,"

* De la Phthisis, p. 215, ed. 2. Paris, 1843.

and "an excessive nervous excitability." Very remarkable "*lesions*;"—let that pass, however. But we cannot let pass the following piece of inductive philosophy, designed for the instruction of youths about, sooner or later, to have on their heads the dreary responsibility of actually treating phthisis:

"We have likewise previously seen, that a diminution in the waste or decomposition of the tissues is equivalent to an increase of supply or growth; and it is a fact, independent of all theory, that a diminution in the amount of respiration will produce this effect. It was on the principle of neutralizing the operation of the inspired oxygen that Dr. Beddoes recommended inhalation of carburetted hydrogen in this disease; and there is no doubt that persons who had been weak and wasted, grew fat and strong under this treatment. Naphtha, creosote, and the non-nitrogenized articles of food, such as starch, gum, and sugar, will act in a similar manner. *So that, you perceive, there are many ways by which we can oppose this first and predisposing lesion of phthisical disease.*" (p. 100.)

Blessed man, says the student to himself; thrice blessed therapeutist! never shall emaciation (or, as my venerated master hath it, "diminished force of growth or reparation") exhibit its withered form in *my* path! with starch, gum, and sugar (no bad things, by the way), a dust of this, and a shake of that, along with a little creosote, I shall drive emaciation before me,—and, please heaven, make my fortune. Now, it is plain that such is the interpretation any reflective student must put on Dr. Evans's doctrine; for a *student* can hardly be expected to see through the loophole of escape which Dr. Evans has so very neatly provided for himself by the simple use of the word "*oppose*:" we may "oppose" emaciation, says the innocent teacher, with the remedies I enumerate. No: had his young Irish auditors caught fully the sense of this little word, they would assuredly have reminded the learned man of the various attempts that have been made from time to time to keep out (or "oppose") the tide with a pitchfork, together with the provoking result, that the tide has hitherto invariably beaten the pitchfork in the long run. After this precious specimen of therapeutics, it seems sufficiently flat to turn to a point of pathology. Dr. Evans calls emaciation "this first and predisposing lesion of phthisical disease:" but what says clinical observation in the hands of Louis? Why, that in *one half* only of the patients observed by him* did loss of flesh appear among the first symptoms,—in *none* is it spoken of as their harbinger.

But Dr. Evans hasn't half done "opposing" yet: hear him in the second lecture again:

"After this rapid review of the lesions which constitute pulmonary consumption, and the means which we possess of baffling them, I think you will already be inclined to agree with me, that there is nothing necessarily fatal or incurable in this disease; *but that, on the contrary, it is just as tractable as other chronic diseases.*" (p. 102.)

Why, this is next door to Professor Holloway's pill and the wafer advertisements of the sham Dr. Locock: the wafers ensure perfect cure of consumption in ten minutes, we believe; so that Dr. Evans may be considered so far to be outdone. We are very sorry for him: but from

* Op et ed. citat. p. 369.

many strange things we see in this strange book, we suppose the defeat is not quite irreparable.

It will probably be expected that we should say something of St. John Long's "treatment" and "nostrum." Dr. Evans tells us concerning the first,—"the principal, or, indeed, to speak more correctly, the sole element in St. John's treatment was counter-irritation." As respects the second (that is, the nostrum), it appears there was none; for, though the turpentine and strong acetic acid liniment was the composition this man-slayer chiefly employed, "we sometimes," says Dr. Evans, "made an alteration in the liniments." It is reported (and, as we believe, correctly) that an individual among the aristocracy, eminent among the eminent for the soundness of scientific judgment characteristic of that branch of the community, gave St. John Long £3000 for his secret! Poor woman, she got the vinegar and turpentine receipt; and may she be happy with it,—the very worst and most uncertain counter-irritant that still holds a place among the long list of such.

Dr. Evans indulges no less in singular words than in singular therapeutical ideas: where did he find the terms "lymphy," "lungy," "enteritai," &c. &c., which occur *passim* in his pages? Where, too (and these examples are jotted down as being the first that catch our eye in turning over the leaves), did he become acquainted with his method of spelling the words "Aritæus" (p. 27); "Cyrrosis" (p. 12, twice); "gum-ammonia" (p. 105); "albuminaria" (p. 121), &c. &c.? But if English orthography fares badly with Dr. Evans, it has no right to complain,—for the French is ten times worse off. The book closes with a professed transcript from Andral's Clinique Médicale, which throughout contains on an average about two errors of spelling *per line*;—there is not a single accent from beginning to end; and we venture to affirm that there is not a Frenchman from Calais to Toulon who could read the extract without real difficulty.

---

